# Editorial

**Published:** 1894-07

**Authors:** 


### Editorial.

In an address recently delivered by Dr. Jacobi before the Eleventh International Congress, at Rome, Italy, he uses these words: "In large cities the thorough, all-around, general practitioner is becoming scarce. Now and then he is expected to be, but the city directory or the agent for the specialists in brain and nerves, in kidneys and appurtenances, in uterus and appendages, in skin and corns, in heart and lungs, in stomach, throat, nose, eyes, ears and what not."

This is strong language but true, and we wish to protest against this state of things and to call upon the profession to bring about a change which will redound to the credit of the physician and the welfare of his patients. Specialism in medicine has accomplished much. In all sciences the evolution of specialists is legitimate and proper, but evolution is a slow process, and the man who expects to achieve success and distinction in a specialty must, before he can treat any organ of the human body, have thoroughly familiarized himself with the organization as a whole, must have knowledge and training, must know and understand the broad principles of physiology and pathology which underlie the successful treatment of all diseased conditions. Before he is competent to treat intelligently and successfully the simplest organ of the human body he should be a *good physician.* The young man who, fresh from his college halls, spends six months or a year in some hospital, or goes abroad and listens for a while to the savants of Europe, and returning to his native town, calls himself a specialist, is not fit to sit at the feet of Æsculapius, and should be driven from the ranks of the profession. Such men are actuated by mercenary motives, and are not worthy to be called physicians. Often they become quacks and charlatans. The public, ever ready to be humbugged, have, without consulting their family physician, flocked to these newly hatched fledgelings, and, as the case may be, have had the nasal septum sawed or cauterized, the uvula clipped, the eyes spectacled, the cervix sewed up, the cervix slit, the uterus curetted, the vagina tamponed, the ovaries removed, and so on, until the patient's pocket is empty or his faith shaken, and he ends where he should have begun, by going to the general practitioner, in other words, to the physician.

Specialists expect and demand greater compensation for their services than the general practitioner. Why? Are they more learned, are they more industrious, are they more conscientious, are they better physicians? We think not. Does it require greater skill and knowledge to remove a cataract, to curette a uterus, to apply the cautery, than to treat intelligently a case of scarlatina, typhoid or diphtheria? Does it involve the worry and vexations, the personal discomfort and danger? The answer is emphatically, *no!*

To entitle the specialist to consideration and patronage at the hands of the public and the profession, he must have brought to bear upon his specialty time and his best efforts; he must acquire skill and knowledge beyond the reach of one working in a broader field; he must know that which is beyond the ken of the masses; then, and not till then, can he honor himself and dignify his calling. There is no more dangerous man than the would-be specialist, galloping at full tilt upon his ever-changing hobby. How many pounds of potassium iodide and the salts of mercury have found their way into the stomachs of non-syphilitic patients; how many healthy ovaries have their resting place in the jars of the gynecologist; how many acres of healthy uterine mucosa have been furrowed by the invading curette, are questions that will go down to posterity unanswered.

---

AMERICAN MEDICAL ASSOCIATION.—Following are the officers for the current year: President, Dr. Donald McLean, of Michigan; Vice-President, Dr. T. C. Loving, of Ohio; Treasurer, Dr. S. P. Newman, of Illinois; Secretary, Dr. William B. Atkinson, of Pennsylvania; Assistant Secretary, Dr. Geo. H. Rohe, of Maryland; Editor of the Association *Journal*, Dr. J. B. Hamilton, of Illinois. The next meeting of the Association will be held in Baltimore, beginning the first Tuesday in May, 1895.

